# In vitro and in vivo anticancer activity of nickel (II) tetraazamacrocyclic diperchlorate complex, [(Ni-Me_8_[14]diene)(ClO_4_)_2_] against ehrlich ascites carcinoma (EAC) and MCF7 cells

**DOI:** 10.1007/s12032-025-02762-w

**Published:** 2025-05-23

**Authors:** Arifur Rahman, Tasfik Ul Haque Pronoy, Kazi Soha, Abdul Auwal, M. Matakabbir Hossain, K. M. Rashel, Md Royhan Gofur, M. Habibur Rahman, Saswata Rabi, Tapashi Ghosh Roy, Nitai Roy, Jahan Ara Khanam, Md Abdur Rakib, Farhadul Islam

**Affiliations:** 1 Department of Biochemistry and Molecular Biology, University of Rajshahi, Rajshahi, 6205 Bangladesh; 2 Department of Veterinary and Animal Sciences, Rajshahi University, Rajshahi, Bangladesh; 3 Department of Chemistry, University of Rajshahi, Rajshahi, 6205 Bangladesh; 4 Department of Chemistry, Faculty of Science, University of Chittagong, Chittagong, 4331 Bangladesh; 5 Department of Biochemistry and Molecular Biology, Patuakhali Science and Technology University, Patuakhali, Bangladesh; 6 School of Medicine and Dentistry, Gold Coast Campus, Griffith University, Queensland, 4222 Australia

**Keywords:** Nickel (II) complex, Tetraazamacrocyclic ligand, Anticancer activity, Cytotoxicity, Toxicity, Apoptosis, EAC cells, MCF7 cells

## Abstract

Cancer remains a global health burden, with a pressing need for more effective treatments. This study uses a novel compound, Nickel (II) diperchlorate complex of the ligand (L): 3,10-C-meso-3,5,7,7,10,12,14,14-octamethyl-1,4,8,11-tetraazacyclotetradeca-4,11-diene, Me_8_[14]diene, designated as [Ni(II)L](ClO_4_)_2_, to explore its potential as an anticancer agent. Its efficacy was evaluated against Ehrlich Ascites Carcinoma (EAC)-bearing Swiss albino mice by monitoring tumor cell growth inhibition, survival time, tumor mass reduction, and hematological profiles. Additionally, cytotoxicity was investigated in vitro using MCF7 breast cancer cells. The apoptotic potential was evaluated through Hoechst staining, with changes in apoptosis-related gene expression (*p53, BCL2, BAX, PARP1, CASP3, CASP8*, and *CASP9*) using RT-qPCR. The test compound’s toxicity was evaluated by monitoring hematological, biochemical, and histological changes. The compound exhibited dose-dependent growth inhibition of EAC cells with 88.45% inhibition at a dose of 200 µg/kg (*p* < 0.01), extended lifespan by 52.63%, reduced tumor weight by 47.83%, and restored hematological parameters in EAC-bearing mice. Cytotoxicity assays yielded LC_50_ and IC_50_ values of 23.73 µg/mL and 71.52 µg/mL, respectively. Apoptosis induction was evidenced by cell membrane blebbing, apoptotic body formation, chromosomal condensation, and nuclear fragmentation in MCF7 cells. Significant upregulation of pro-apoptotic genes such as *p53, BAX, PARP1, CASP3, CASP8*, and *CASP9*, alongside downregulation of anti-apoptotic gene *BCL2,* implied activation of the apoptotic pathway in cancer cells, followed by compound treatment. Moreover, no long-term negative impacts on tissue levels or hematological or biochemical markers were noted in the mice. Altogether, [Ni(II)L](ClO_4_)_2_ demonstrates promising anticancer activity and could serve as a potential chemotherapeutic agent, pending further studies.

## Introduction

Cancer remains a leading global health concern, with over 20 million new cases and nearly 9.7 million deaths reported worldwide in 2022, according to the American Cancer Society (ACS) [1]. This rising prevalence of cancer underscores an urgent need for novel cancer therapies. Cancer is a cellular disease marked by uncontrolled cell proliferation, apoptosis evasion, tissue invasion, and metastasis. It disrupts normal cell division and programmed cell death [2]. Conventional treatments such as surgery, radiation therapy, chemotherapy, and many other therapies have limitations, including adverse side effects and drug resistance. Thus, identifying more effective, targeted, and less toxic chemotherapeutic agents is a critical scientific priority.

Metal-based compounds have gained attention in cancer therapy since the 1960 s, following the discovery of cisplatin’s anticancer activity [3–5]. Metal complexes with platinum, ruthenium, gold, rhodium, silver, cobalt, zinc, copper, and nickel have shown potential chemotherapeutic activity, including apoptosis induction and tumor growth inhibition [6]. These effects are attributed to metals’ distinctive properties, including redox activity, coordination modes, DNA interaction, reactive oxygen species (ROS) generation, and modulation of signaling pathways associated with cell proliferation and apoptosis [6]. For example, platinum-based drugs like oxaliplatin, carboplatin, and nedaplatin induce apoptosis by developing DNA lesions, interrupting DNA duplication, suppressing RNA synthesis, and inducing immune responses [7, 8]. In contrast, copper-based Topoisomerase-1 (Top1) inhibitors such as Plumbagin-Cu(II) and phenanthroline-Cu(II) complex induce apoptosis via mitochondrial signaling pathways [9, 10]. Ruthenium(III) complexes consisting of triazolopyrimidine, phenanthroline, and polypyrimidine ligands have exhibited potent cytotoxicity against primary tumors, particularly those that are resistant to cisplatin [11]. Silver complexes of N-heterocyclic carbenes, 5-fluorouracil, NSAIDs, phosphines, carboxylates, Schiff bases, and dehydronorcantharidin exhibit anticancer effects through mechanisms such as DNA binding, topoisomerase inhibition, ROS generation, and apoptosis induction [12]. Similarly, coordination complexes of gold, such as Au(I) thiosugar complexes, Au(III) complexes of 2-[(dimethylamino)methyl]phenyl ligands, and auranofin analogs have demonstrated cytotoxic activity against a variety of cancer cells [13].

Recent studies have highlighted nickel complexes as potential anticancer agents, showing potent cytotoxic effects and DNA cleavage activity in cancer cells. Several nickel-based compounds such as Nickel(II) N-(2-hydroxyacetophenone) glycinate (NiNG) and nickel(II)-bis (thiosemicarbazone) showed considerable efficacy in multidrug resistance that involves reactive oxygen species (ROS)-mediated redox imbalance for regulation of caspase-3-dependent apoptosis against human breast (MCF7) and lung (A549) cancer cells [14, 15]. Despite these promising findings, Ni-based compounds remain underexplored compared to Pt, Au, Pd, Ru, or Cu-based compounds.

Tetraazamacrocyclic metal complexes show significant antioxidative properties due to substantial radical scavenging potency, greater binding constancy with double-strand DNA, and antibacterial activity [16, 17]. Given these findings, this study focuses on a Nickel (II) diperchlorate complex of the ligand (L): 3,10-C-meso-3,5,7,7,10,12,14,14-octamethyl-1,4,8,11-tetraazacyclotetradeca-4,11-diene, Me_8_[14]diene, designated as [Ni(II)L](ClO_4_)_2_ to evaluate its cytotoxicity, anticancer activity, and toxicity. The assessment includes in vivo and in vitro studies to determine its potential as an effective chemotherapeutic agent, with specific attention to cell growth inhibition, apoptosis induction, gene expression, and safety profile.

## Materials and methods

### Chemicals and reagents

The chemicals and reagents were of analytical and molecular biology grade used in the current study. RPMI 1660 cell culture media and Trypan blue stain (Thermo Fisher Scientific, Waltham, MA USA). Reverse transcription system and master mix (Promega Madison, Wisconsin, USA), RNA extraction kit (Favorgen, Ping Tung, TAIWAN), PCR system (Roche, Basel, Switzerland), Biochemistry test kits (Human, Louisville, USA), Methanol, Ethanol, chloroform, EDTA, DMSO, PBS, Isopropanol, and other laboratory regents (Merck, Rahway, NJ, USA).

### Animal model

In this study, adult male Swiss albino mice (20–23 g) were selected. These mice were bred in the Animal House facility at the Department of Biochemistry and Molecular Biology, University of Rajshahi, Bangladesh. They were housed under controlled environmental parameters (temperature 25–32 °C, humidity 30–70%, 14-h light/10-h dark cycle), wood shavings were used as bedding, loud or high-frequency sounds were avoided, and cages were cleaned with bedding changed twice weekly to ensure consistency and animal welfare. This study was performed in line with the principles of the Declaration of Helsinki. Approval was granted by the Ethics Committee of the University of Rajshahi, Bangladesh (18-08-2021/No. 293(13)/320-IAMEBBC/IBSc). Specific ethical guidelines were followed as per the ARRIVE guidelines, enhancing transparency in animal care practices.

### Tumor cell line

EAC cells, provided by the Indian Institute for Chemical Biology (IICB), in Kolkata, India, were maintained in our lab by periodic intraperitoneal inoculation of mice at 10^5^ cells every two weeks. Additionally, the MCF7 human breast cancer cell was obtained from the American Type Culture Collection (ATCC) and cultured according to the protocol used by Kabir et al. [18]. Culture conditions were optimized using RPMI-1640 medium supplemented with 10% FBS (Fetal Bovine Serum), 1% Penicillin–Streptomycin, and 2 mM L-Glutamine, ensuring an ideal environment by incubating at 37 °C with 5% CO₂ and 70–80% cell passing confluency using 0.25% Trypsin–EDTA for maintaining healthy growth.

### Synthesis and characterization of Ni(II)-complex

The compound, [Ni(II)L](ClO_4_)_2_, was synthesized and characterized as described in a previous report [19].

### ***Brine shrimp lethality bioassay of [Ni(II)L](ClO***_***4***_***)***_***2***_

The cytotoxicity of the test compound was tested on Artemia salina in seawater at concentrations (2 µg/mL, 4 µg/mL, 6 µg/mL, 8 µg/mL, 10 µg/mL, and 15 µg/mL) for 24 h. LC_50_ values were determined by regression analysis. This assay is a cost-effective preliminary test for assessing cytotoxicity and bioactivity, serving as an initial screening tool before more complex mammalian studies [20].

### MTT assay

The cytotoxicity of the test compound against the MCF7 breast cancer cell line was assessed using the MTT colorimetric assay [21]. 1 × 10^6^ MCF7 cells in 200 μL RPMI-1640 medium were seeded into each well of a 96-well plate and exposed to five concentrations of the test compound (12.5, 25, 50, 100, and 200 μg/mL). Untreated MCF7 cells in DMSO served as a control. The experiment was conducted in triplicate to minimize experimental variability. Following a 24-h incubation at 37 °C in a CO_2_ incubator, aliquots were removed, and each well received MTT and PBS, respectively. After an additional 8-h incubation at 37 °C, acidic isopropanol was added, followed by another hour of incubation. The absorbance was then measured at 570 nm using a microtiter plate reader (Optica Microplate Reader, Mikura Ltd., Horsham, UK).

### *Inhibition of EAC cell growth *in vivo

Tumor cell growth inhibition was assessed in vivo following a standard protocol [22]. The study included three groups of mice (6 mice per group), each receiving an inoculation of 1.6 × 10^6^ EAC cells per mouse intraperitoneally on the first day. The treatment began 24 h after tumor inoculation and continued for 5 days. Mice in groups 2 and 3 received the test compound at doses of 100 µg/kg and 200 µg/kg of body weight per day, respectively, via intraperitoneal injection (0.1 mL per injection). Group 1 served as a control and received normal saline intraperitoneally. On the sixth day, each group of mice was sacrificed, and peritoneal fluid was collected using 0.9% saline. The number of viable tumor cells in the ascitic fluid was counted by a hemocytometer using trypan blue staining. Cell growth inhibition was calculated using the formula:

$$\% {\text{ Cell growth inhibition}}\, = \,\left( {1 - {\text{ T }}/{\text{ C}}} \right)\, \times \,100$$

where *T* = Mean viable tumor cell count in treated groups and *C* = Mean viable tumor cell count in control group.

### Average tumor-associated weight gain and survival time

Survival time and tumor burden were assessed following a protocol adapted from previous publications [23]. Mice were divided into three groups (6 mice per group) and inoculated with 1.6 × 10^6^ EAC cells per mouse via intraperitoneal injection on the first day. The control group (group 1) received normal saline. After 24 h of post-inoculation, intraperitoneal treatment with the test compound was administered to Groups 2 and 3 at doses of 100 µg/kg and 200 µg/kg of body weight, respectively, for 10 consecutive days. Body weight was measured every 48 h, extending up to 25 days from day zero. The tumor-associated weight gain was used as an indirect measure of tumor burden, based on the accumulation of ascitic fluid. Survival was monitored daily, and the following parameters were calculated:

$${\text{Mean Survival Time }}\left( {{\text{MST}}} \right)\, = \,\left( {\Sigma {\text{ Survival days for each mouse}}} \right) \, /{\text{ Total mice}}.$$

$$\% {\text{ Increase in Life Span }}\left( {{\text{ILS}}} \right)\, = \,\left( {\left( {{\text{MST of treated group }}/{\text{ MST of control group}}} \right){-}{1}} \right)\, \times \,{1}00.$$

$$\% {\text{ Reduction in Tumor}} - {\text{Associated Weight Gain }}\left( {{\text{RTW}}} \right)\, = \,\left( {\left( {{\text{Weight gain in control}}{-}{\text{weight gain in treated}}} \right) \, /{\text{weight gain in control}}} \right)\, \times \,{1}00.$$

### Hematological profile

The effect of the test compound on hematological parameters, including white blood cells (WBC), red blood cells (RBC), and hemoglobin (Hb) was investigated using a standard method [24], involving cell dilution fluids and a hemocytometer. Four groups of mice were included in the study: one group was normal (non-EAC-bearing normal mice), one served as a control group (EAC-bearing mice without treatment), and the remaining two groups were EAC-induced and treated with the test compound at doses of 100 µg and 200 µg per kg body weight per day, administered intraperitoneally for 10 days. Each group consisted of six mice. On day zero, EAC cells (0.1 mL, 1.6 × 10⁶ cells/mouse) were administered intraperitoneally into EAC-bearing groups. Following a 24-h inoculation period, the test chemical was administered regularly. On the 11 th day after inoculation, blood samples were taken via tail puncture into anticoagulant-containing tubes, and hematological parameters (WBC, RBC, and Hb levels) were analyzed to determine the test compound’s effectiveness.

### Morphological appearance and nuclear damage

The induction of apoptosis in MCF7 cells by the test compound was evaluated by observing morphological changes and nuclear damage under a fluorescence microscope (Olympus IX71, Seoul, Korea) [25]. Briefly, 96-well plates were seeded with cancer cells (5 × 10^5^ cells/well) and cultured for 24 h at 37 °C in an incubator with 5% CO_2_. Following the incubations, cells were exposed to the test compound at different concentrations (10–100 µg/mL) and then incubated for an additional 24 h under the same conditions. The cells were then stained with Hoechst 33,342 (0.1 μg/mL) at 37 °C for 20 min, washed and resuspended in phosphate buffer saline (PBS), and examined for morphological changes indicative of apoptosis, including nuclear damage.

### mRNA extraction and cDNA synthesis

To evaluate the expression levels of apoptosis-regulating genes in MCF7 cells treated with test compound (200 µg/mL), RNA was extracted from both treated and untreated MCF7 cells using “FavorPrep™ Total RNA Isolation Kit (Favorgen, Taiwan)” followed by the manufacturer’s guidelines. The extracted RNA concentrations from treated (270.7 ng/µl) and control (193.7 ng/µl) MCF7 cells were determined by the Nanodrop One spectrophotometer and by measuring absorption ratios (A_260/280_ and A_260/230_) to ensure high quality RNA before cDNA synthesis. The RNA structural integrity of the RNA was further assured through 1.8% agarose gel electrophoresis. The complementary DNA (cDNA) was synthesized from the RNA samples using the GoScript™ Reverse Transcription System kit (PROMEGA) followed by the manufacturer’s guidelines. The synthesized cDNA was stored at −20 °C for subsequent analysis.

**Table 1:** List of Primers

Gene	Forward primer sequence (5ʹ–3ʹ)	Reverse primer sequence (5ʹ–3ʹ)
p53	GCC CAA CAA CAC CAG CTC CT	CCT GGG CAT CCT TGA GTT CC
BAX	CCCGAGAGGTCTTTTTCCGAG	CCAGCCCATGATGGTTCTGAT
PARP1	GGCCTCGGTGGATGGAATG	GCAAACTAACCCGGATAGTCTCT
CASP3	CATGGAAGCGAATCAATGGACT	CTGTACCAGACCGAGATGTCA
CASP8	ACA CAG TCG AGT AGA CTC TCAAA	AGG AAG TGA TGC TCG TTC AGA
CASP9	CTG TCT ACG GCA CAG ATG GAT	GGG ACT CGT CTT CAG GGG AA
BCL2	GGTGGGGTCATGTGTGTGG	CGGTTCAGGTACTCAGTCATCC
GAPDH	GGAGCGAGATCCCTCCAAAAT	GGCTGTTGTCATACTTCTCATGG

### Reverse transcription polymerase chain reaction (RT-PCR)

The expression of key apoptosis regulatory genes such as p53, BAX, BCL2, PARP1, CASP3, CASP8, and CASP9 was analyzed in MCF7 cells treated with the test compound (200 µg/mL) using RT-PCR [26]. The cDNA was used as PCR template and GAPDH served as the housekeeping control. The sequences of primers against examined genes are shown in Table 1. RT-PCR was conducted using a Light cycler 96 PCR system (ROCHE, 4414 Lake Boone Trail. Raleigh, NC 27607, USA). In brief, each 20 µL PCR reaction mixture contained 10 µL of master mixture (Promega, Madison, WI, USA), 2 µL of template cDNA (50 ng), 1 µL of each forward and reverse primers, and 6 µL of nuclease-free water. Amplification involved an initial denaturation at 95 °C for 10 min, followed by 45 cycles of 15 s at 95 °C for denaturation and 1 min at 60 °C for annealing and extension. Relative expression levels of the target genes were calculated using the comparative Ct method (ΔΔCt), normalizing to GAPDH as the endogenous control. Melting curve analysis, using a temperature gradient from 60 °C to 95 °C at 0.3 °C increments, was utilized to confirm the amplification products’ melting temperatures and identify primer dimers.

## Toxicological study of [Ni(II)L](ClO_4_)_2_

### Monitoring of biochemical parameters

The biochemical parameters of blood were estimated following the established protocol as documented in prior research [24]. In this study, two groups of male Swiss albino mice, each consisting of nine mice, were designated for the experiment. One group served as the control, comprising normal mice, while the other groups received an intraperitoneal administration of the test compound at a dosage of 200 µg/kg body weight. The objective was to evaluate various biochemical parameters, including serum glutamic pyruvic transaminase (SGPT), serum glutamic oxaloacetic transaminase (SGOT), serum glucose, creatinine, and cholesterol levels. In this assay, three mice from each group were sacrificed, and blood was drawn from the heart into 2 mL plastic centrifuge tubes on days 10, 15, and 25. The blood samples underwent incubation at room temperature for one hour to facilitate clotting, after which clear straw serum was obtained through centrifugation at 4000 rpm for 10 min, utilizing the “WIFUNG centrifuge LABOR-50 M.” The semi-automated biochemistry analyzer “Humalyzer 3000” was employed to ascertain the specified biochemical parameters.

### Monitoring of hematological profile

To determine the effect of test compound on hematological abnormalities such as WBC, RBC, and Hb content, two groups of Swiss albino mice were used as animal models (9 mice in each group), group 1 as control (normal mice) and group 2 as treatment (treated with 200 µg/kg body weight/day dose (*i.p.)* of test compound for 10 days). Blood samples were collected from three mice of each group by tail puncture in anti-coagulant-containing tubes on days 10, 15, and 25. The hematological parameters were determined by the standard published methods [24] using cell dilution fluids, Hellige Sahli’s hemometer, and hemocytometer.

### Histological study

To determine the effects of the test compound in the tissue section, histology of major organs like the heart, lung, liver, kidney, and spleen was performed to observe any changes like inflammation, degradation, regeneration, congestion, infiltration, etc. The study was conducted with two groups of Swiss albino mice, each containing three mice, including a control group (normal mice) and a treatment group treated with 200 µg/kg of body weight of test compound for 10 consecutive days. The organs were collected on the 25 th day and tissue pieces were preserved in 10% formal saline and processed by employing routine paraffin embedding method [24]. The Sections of five-micron thickness were cut and stained by the Hematoxylin and Eosin (H&E) method to prepare slides [27]. The slides were viewed under the Motic Advanced system microscope (B, series) using Motic J. 1 software.

### Statistical analysis

Data (percent of cell growth inhibition, increase of life span, body/tumor weight, biochemical parameters, hematological profile, and apoptosis regulatory gene expression) are expressed as mean ± SEM (Standard Error of Mean). Data has been analyzed with Welch’s test (EAC cell growth inhibition, survival time, hematological parameters, gene expression), nonparametric paired T-test (average tumor weight, biochemical parameters), and ordinary one-way (ANOVA) (MCF7 cell growth inhibition) using GraphPad Prism 8 software. Where *P* < 0.05 is considered to be statistically significant.

## Results

### ***Cytotoxicity of [Ni(II)L](ClO***_***4***_***)***_***2***_

**Fig. 1:**
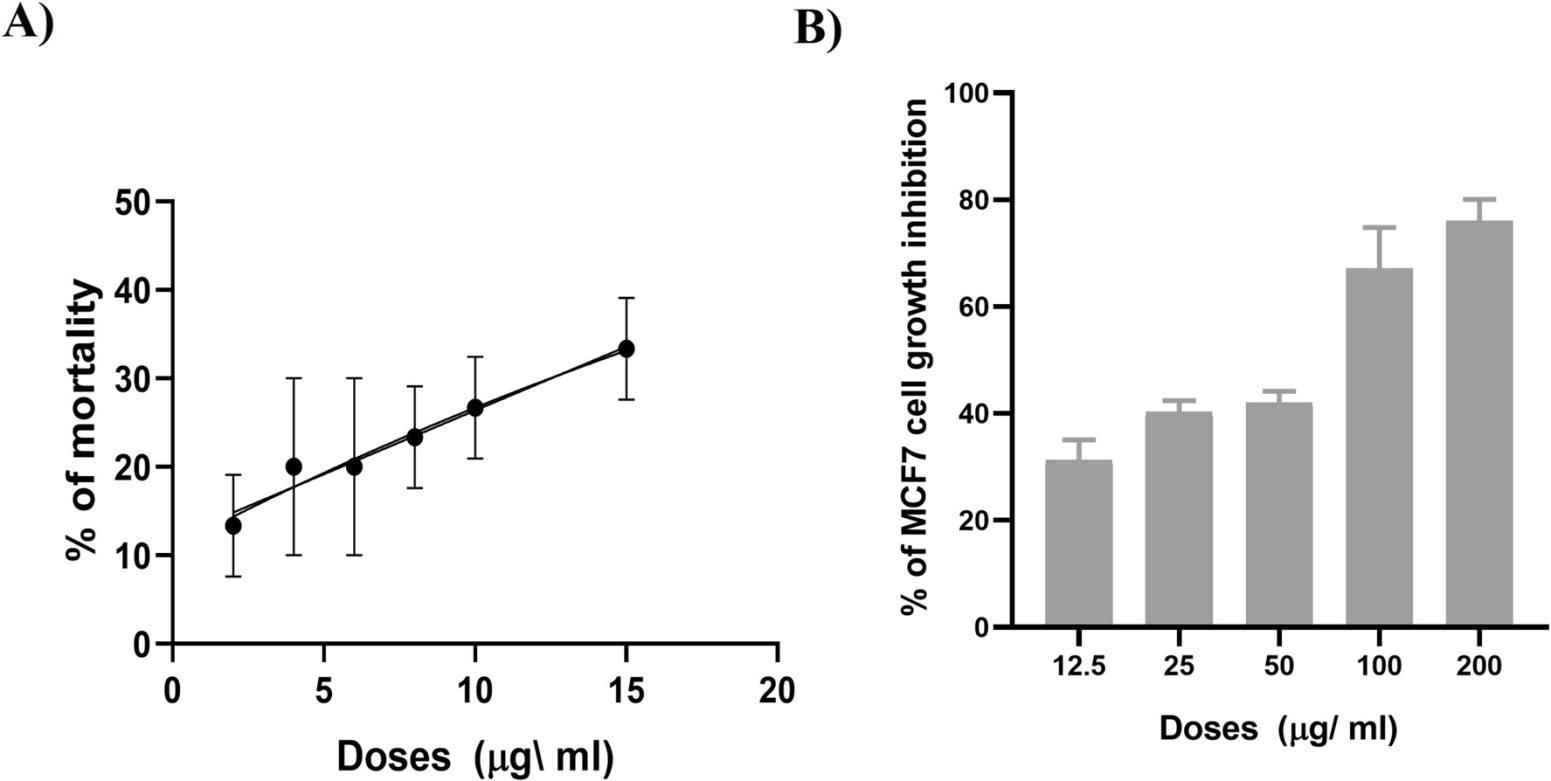
In vitro cytotoxicity of the complex. **A** Effect of the test compound on the mortality of brine shrimp nauplii. **B** Effect of test compound at five different doses on MCF7 cell growth inhibition. The results are presented as mean ± SEM. The significant value is ***P < 0.0001, compared to the control (DMSO-treated only), analyzed by ordinary one-way ANOVA using GraphPad Prism 8 software

The cytotoxic impact of the test compound was assessed using the brine shrimp lethality bioassay. The medium lethal concentration (LC_50_) for brine shrimp lethality was determined to be 23.73 µg/mL using the linear regression equation (*y* = 1.4419 × x + 11.964). The concentration of the test compound was found to be correlated with an increase in the percentage mortality of nauplii, as illustrated in Fig. 1A.

### Inhibition of MCF7 cell growth

The cytotoxic effect of the test compound against the MCF7 breast cancer cell line was assessed using the MTT bioassay. Compared to the DMSO-treated MCF7 cells (control), the corresponding cell growth inhibition rate was observed to be 31.34%, 40.35%, 42.06%, 67.21%, and 76.12% after 24 h of exposure to the test compound at doses of 12.5, 25, 50, 100, and 200 µg/mL. This increased significantly in a dose-dependent manner, as illustrated in Fig. 1B. Using the linear regression equation (*y* = 0.2377 × x + 33.00), the IC_50_ value was calculated to be 71.52 µg/mL.

### *Inhibition of EAC cell growth *in vivo

**Fig. 2:**
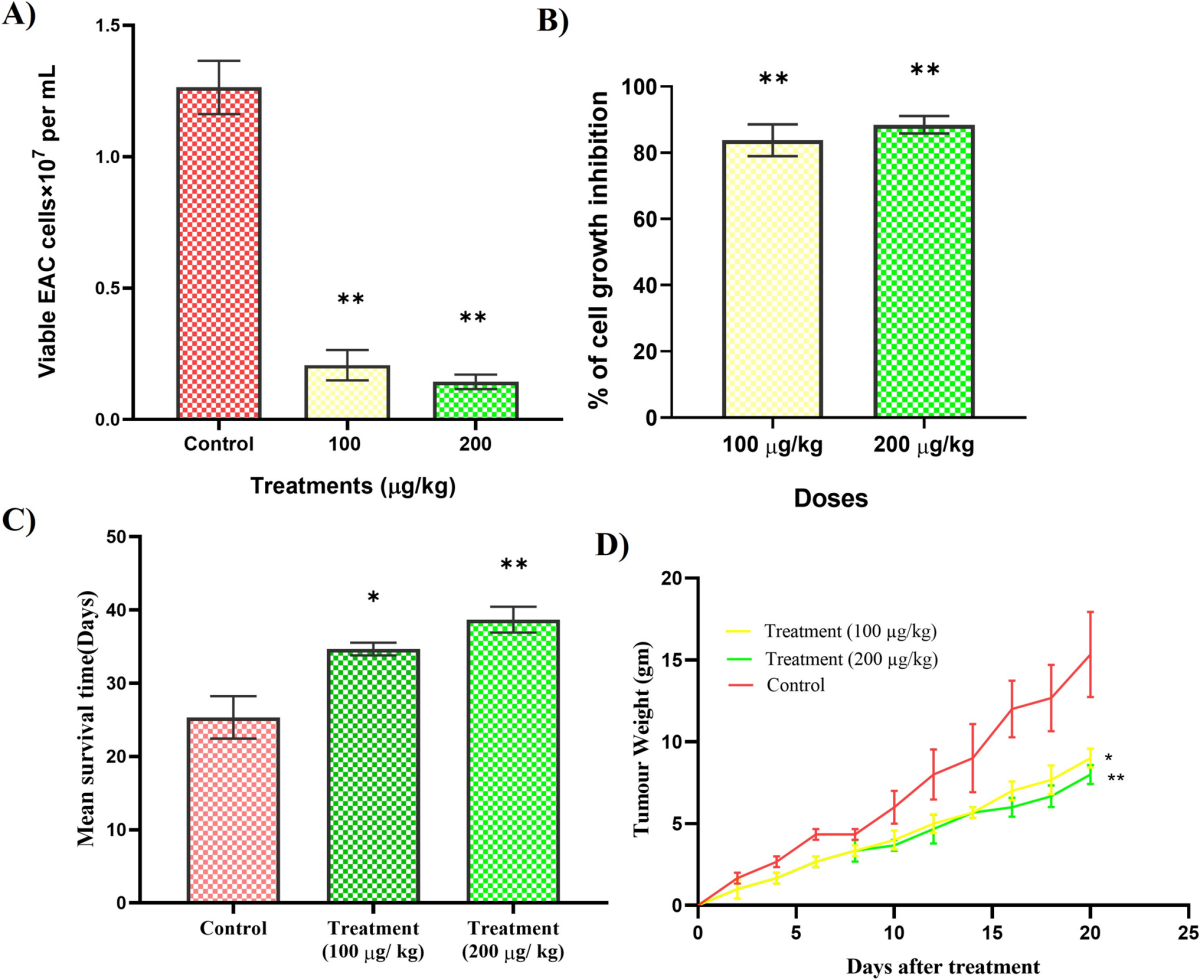
In vivo anticancer activity of test compound. **A** Number of viable EAC cells, **B** percentage of EAC cell growth inhibition. **C** Effects of the test compound on survival time of EAC-bearing mice. **D** Effect of the test compound on tumor-associated weight gain of EAC-bearing mice. The results are presented as mean ± SEM. Significant value is indicated by **P < 0.01 when compared to the control group (EAC-bearing only), analyzed by Welch’s test using GraphPad Prism 8 software

In vivo ascitic tumor cell growth inhibition was observed are shown in Fig. 2A and B. The test compound significantly reduced the number of viable tumor cells in the ascitic fluid (Fig. 2A). The highest cell growth inhibition (88.45%) was found with the 200 µg/kg/day dosage compared with the control group (untreated EAC-bearing only) (*p* < 0.01), while test compound at 100 µg/kg/day doses inhibited 83.78% cell growth (*p* < 0.01) (Fig. 2B).

### Average tumor-associated weight gain and survival time

Figure 2C illustrates the impact of the test compound on the survival duration of EAC-bearing mice. The administration of the test compound at doses of 200 µg and 100 µg/kg/day/mouse (i.p.) resulted in a significant increase in lifespan, with enhancements of 52.63% (*p* < 0.01) and 36.84% (*p* < 0.05), respectively, when compared to the control group of untreated EAC-bearing mice (Fig. 2C). Figure 2D illustrates the impact of the test compound on the average tumor-associated body weight gain used as a surrogate marker for tumor burden. The control group, consisting solely of untreated EAC-bearing subjects, exhibited a 63.88% increase in body weight by day 20. In contrast, mice administered the test compound at doses of 100 µg and 200 µg/kg/day/mouse (i.p.) demonstrated increases of only 38.57% (*p* < 0.05) and 31.58% (*p* < 0.01), respectively, on day 20 (Fig. 2D). The findings indicated that the administration of test compound at doses of 100 µg and 200 µg/kg/day/mouse led to a significant reduction in tumor-associated weight gain, with decreases of 41.30% and 47.83%, respectively, when compared with the control group (untreated EAC-bearing only) (Fig. 2D).

### Hematological profile

Throughout the progression of the tumor, alterations in hematological parameters from their baseline levels were observed. Only the EAC-bearing mice demonstrated a reduction in Hb levels (% of Hb) and a diminished count of RBC when contrasted with normal mice, whereas the total WBC showed an increase. The administration of the test compound at doses of 100 µg and 200 µg/kg/day/mouse (i.p.) resulted in a moderate normalization of hematological parameters, demonstrating a dose-dependent effect, as illustrated in Table 2.

**Table 2:** Effects of the test compound on hematological parameters in normal and EAC-bearing mice on day 12 of tumor inoculation

Name experiment	RBC cells/mL	WBC cells/mL	% of Hb gm/dL
Normal Mice	(6.05 ± 0.09) × 10^9^	(8.67 ± 0.88) × 10^6^	7.5 ± 0.32
Control EAC-bearing mice	(4.19 ± 0.09) × 10^9^	(37.7 ± 1.76) × 10^6^	5.3 ± 0.21
EAC- bearing mice + Nickel (II) complex (100 µg/kg)	(4.77 ± 0.07) × 10^9^ **	(33.3 ± 2.40) × 10^6^	6.0 ± 0.12
EAC- bearing mice + Nickel (II)complex (200 µg/kg)	(4.99 ± 0.12) × 10^9^ **	(28.3 ± 1.76) × 10^6^*	6.6 ± 0.10 *

The results are presented as mean ± SEM. Significant values are indicated by *P < 0.05 and **P < 0.01 when compared to the control groups (EAC-bearing only), analyzed by Welch’s test using GraphPad Prism 8 software

### Morphological appearance and nuclear damage

**Fig. 3:**
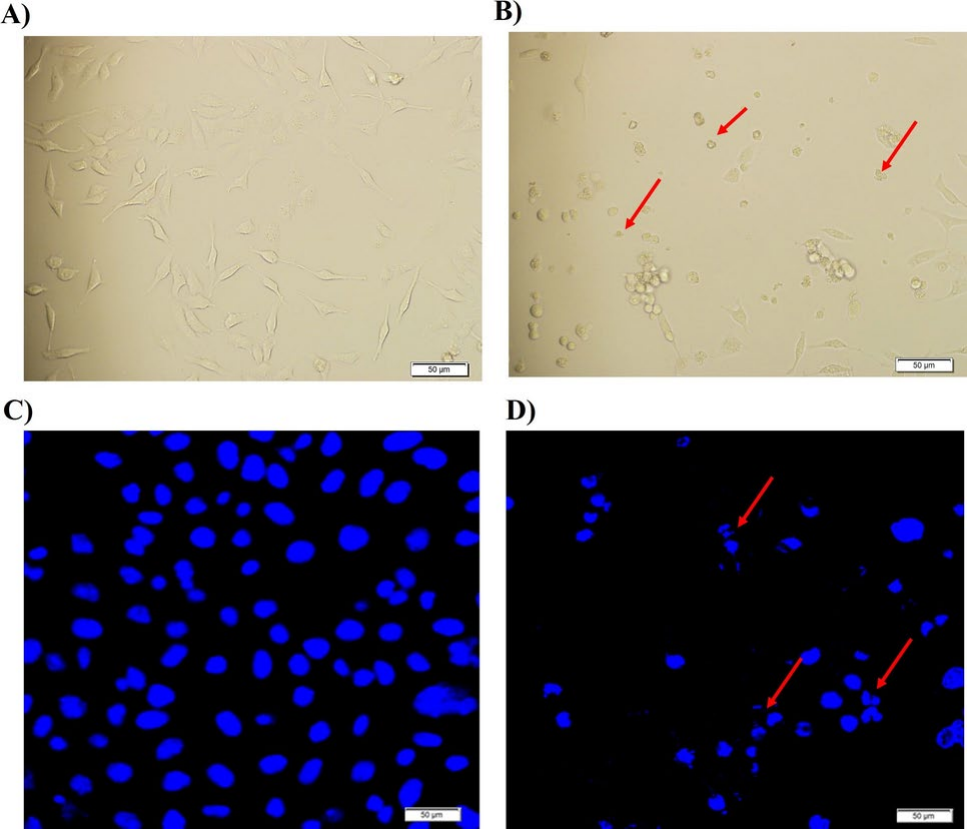
Effect of the test compound on MCF7 cell morphology. **A** Control MCF7 cells under a phase-contrast microscope. **B** Treated cells under a phase-contrast microscope. **C** Control MCF7 cells under a fluorescence microscope. No apoptotic body formation. **D** MCF7 cells were treated with the complex under a fluorescence microscope. The red arrows indicated the apoptotic body formation. Scale bar 50 µm

Cancer cells (MCF7) in the control group showed regular, round, and uniform nuclear stains under a fluorescence microscope followed by Hoechst 33,342 staining (Fig. 3A). On the other hand, cells treated with the compound (200 µg/mL) exhibited apoptotic features such as membrane blebbing, and condensed and fragmented nuclear material (Fig. 3B). Similarly, under a phase-contrast microscope, control MCF7 cells did not exhibit any apoptotic body formation, while cells receiving the complex treatment showed apoptotic body formation (Fig. 3C and D).

### Gene expression analysis

**Fig. 4:**
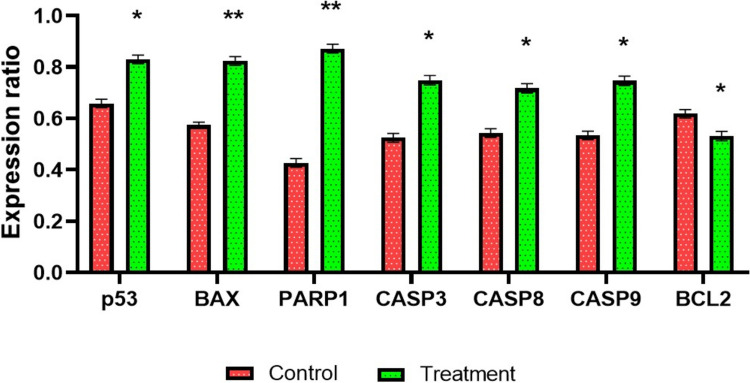
Effect of the test compound on apoptosis regulatory gene expression in MCF7 cells. Treatment of MCF7 cells with the complex caused activation of pro-apoptotic genes such as p53, BAX, Cas-3, −8, −9, whereas inactivate anti-apoptotic Bcl2 gene. The complex also promotes the expression of PARP1 expression in MCF7 cells. The results are presented as mean ± SEM. Significant values are indicated by *P < 0.05 and **P < 0.01 when compared to the control group (MCF7 cells), analyzed by Welch’s test using GraphPad Prism 8 software

The investigation into the apoptosis-inducing property of the test compound involved an examination of the expression levels of p53, BAX, PARP1, CASP3, CASP8, CASP9, and BCL2 in both the control group (MCF7 cells) and the treatment group (MCF7 cells treated with the test compound at a dosage of 200 µg/mL). The results demonstrated that the MCF7 cells subjected to the test compound showed a significant (*p* > 0.05) increase in the expression of pro-apoptotic genes, including p53, BAX, PARP1, CASP3, CASP8, and CASP9, alongside a decrease in the expression of the anti-apoptotic gene BCL2 when compared to the control group of untreated MCF7 cells (Fig. 4). The analysis of data was conducted using RT-PCR ΔΔ CT (Cycle Threshold) values, with normalization performed against the GAPDH housekeeping gene.

## Toxicological study of [Ni(II)L](ClO_4_)_2_

### Effect on biochemical parameters

**Fig. 5:**
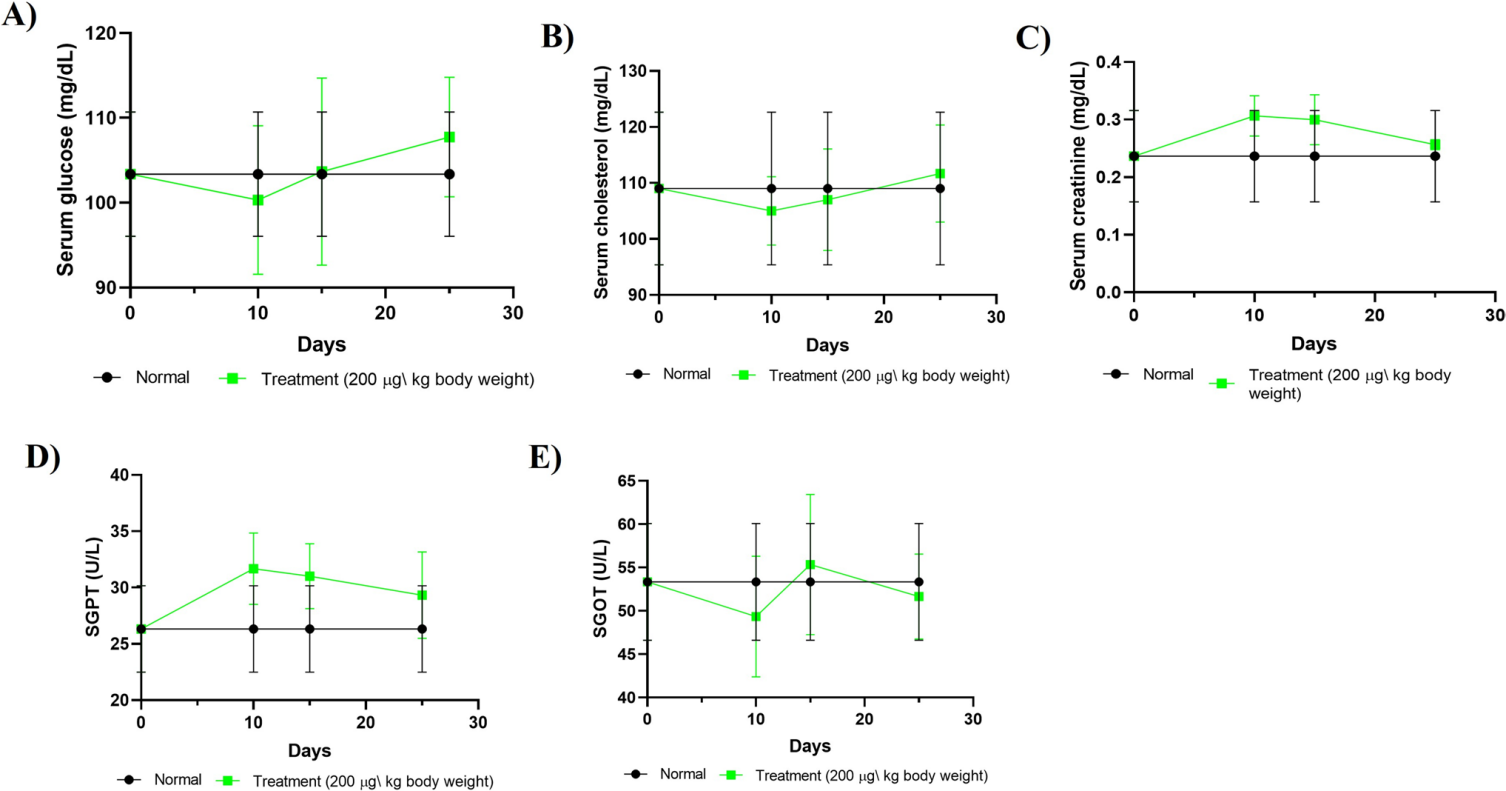
Effect of the test compound on biochemical parameters on days 10, 15, and 25. Treatment of normal Swiss albino mice with the test compound for ten consecutive days did not show any significant alteration of key biochemical parameters such as glucose, creatinine, cholesterol, SGPT, and SGOT on days 10, 15, and 25. The results are presented as mean ± SEM. There is no statistically significant value between control (normal mice) and treatment groups, Where *P* < 0.05 was considered to be statistically significant, analyzed by a nonparametric paired T-test using GraphPad Prism 8 software

The impact of the test compound on biochemical parameters, including glucose, creatinine, cholesterol, SGPT, and SGOT, was assessed by comparing normal mice with those treated with the test compound at a dosage of 200 µg/kg body weight/day/mouse (i.p.) over a span of 10 consecutive days. Observations were made on days 10, 15, and 25, as illustrated in Fig. 5. The observation indicated a gradual increase in the levels of creatinine and SGPT throughout the treatment period, with a slight normalization noted on day 25. Furthermore, no significant changes were observed in other parameters, including glucose, cholesterol, and SGOT, when comparing the control group (normal mice) to the treatment groups.

### Effect on hematological profile

The impact of the test compound on hematological parameters in Swiss albino mice, administered at a dosage of 200 µg/kg body weight/day/mouse (i.p.) over 10 consecutive days, is presented in Table 3 on days 10, 15, and 25. The observation indicated a moderate increase in the WBC value during the administration of the test compound on day 10, followed by a gradual decrease after the cessation of treatment on days 15 and 25. Nonetheless, no significant alterations were observed in the RBC and hemoglobin values when compared to the normal and treatment groups of mice (Table 3).

**Table 3:** Effects of Nickel (II) tetraazamacrocyclic diperchlorate complex, [(NiL)(ClO_4_)_2_] on blood parameters on days 10, 15, and 25

Name of experiment	Days	RBC cells/mL	WBC cells/mL	% of Hb gm/dL
Normal Mice	–	(6.05 ± 0.09) × 10^9^	(8.67 ± 0.88) × 10^6^	7.5 ± 0.32
Normal mice + Nickel (II) complex (200 µg/kg)	10	(5.95 ± 0.06) × 10^9^	(27.67 ± 4.37) × 10^6^ *	7.1 ± 0.47
	15	(6.18 ± 0.03) × 10^9^	(26.00 ± 2.31) × 10^6^ **	7.8 ± 0.31
	25	(6.38 ± 0.04) × 10^9^	(21.33 ± 3.52) × 10^6^	8.1 ± 0.37

The results are presented as mean ± SEM. Significant values are indicated by *P < 0.05 and **P < 0.01 when compared to the control groups (Normal mice), analyzed by Welch’s test using GraphPad Prism 8 software

### Effect on histology

**Fig. 6:**
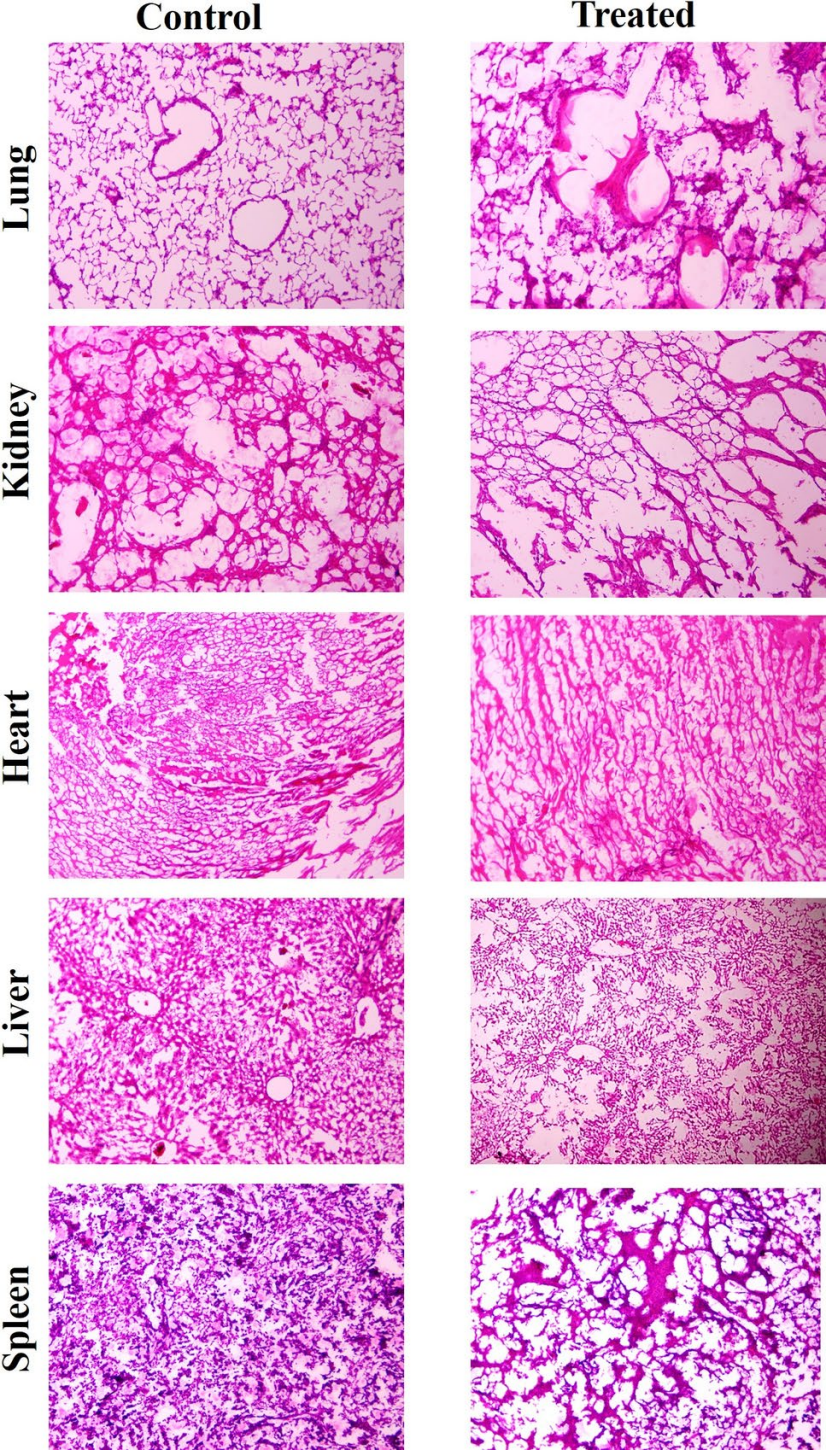
Effect of the test compound on tissue level of major organs of Swiss albino mice. The mice were treated with [(NiL)(ClO_4_)_2_] complex for 10 consecutive days at a dose of 200 µg/kg of body weight/mice/day. Histological analysis of the tissue sections did not exhibit any notable changes (degeneration/regeneration) in the tested organs of the animals

Histological analysis of tissues of major organs (*e.g.,* liver, kidney, lungs, spleen, and heart) of control and mice receiving the complex treatment for a consecutive 10 days showed no major damages (degeneration, regeneration etc.,) in comparison to that of control mice. The treatment only induces minor deformation of the animal organs (Fig. 6).

## Discussion

The identification of potent anticancer agents stands as a critical focus within the field of medical research. This manuscript presents the evaluation of anticancer activity of [Ni(II)L](ClO_4_)_2_. The study is motivated by the encouraging therapeutic potential that metal-based complexes hold in the field of oncology. Previous research has underscored the effectiveness of various metals, including platinum, ruthenium, gold, rhodium, copper, and nickel, in promoting apoptosis and suppressing tumor growth, thereby, establishing them as strong contenders in the realm of cancer therapy. The exploration of azamacrocyclic metal complexes reveals noteworthy pharmacological activities, particularly their antioxidant and antibacterial properties, which underscore their potential as multifunctional agents in therapeutic applications. The study expands upon these findings by systematically examining the cytotoxicity, induction of apoptosis, in vivo anticancer efficacy, and toxicity of [Ni(II)L](ClO_4_)_2_, which has produced promising results.

In this study, we performed the cytotoxicity screening assay of [Ni(II)L](ClO_4_)_2_ against Artemia salina (brine shrimp) and MCF7 breast cancer cell line (in vitro). Cytotoxicity is considered as the effect of a compound to induce cell death by apoptosis or necrosis [28]. The study found LC_50_ for brine shrimp lethality was 23.73 µg/mL and IC_50_ for inhibition of MCF7 cell growth was 71.52 µg/mL; indicating the compound’s significant effect on inducing cell death. In comparison with other metal-based drugs such as platinum-based (cisplatin) exhibits IC_50_ (9–56 µM/2.70–16.81 µg/mL) in various cancer cell lines with several side effects like nephrotoxicity and neurotoxicity [29, 30], Ruthenium-based compounds exhibit IC_50_ (9–191 µM/8–175 µg/mL) for MCF7 breast cancer cells [31, 32], copper-based complexes show IC50 (32–104 µg/mL) [33, 34], suggest a favorable cytotoxic profile of ([Ni(II)L](ClO_4_)_2_.

The anticancer activity of [Ni(II)L](ClO_4_)_2_ has been studied against EAC cell line in vivo using Swiss albino mice as the experimental animal model. This study found an effective EAC cell growth inhibition, an increase in life span, and inhibition of tumor weight of EAC-induced treated mice compared with the control group (non-treated mice). These three parameters of in vivo investigations are crucial indicators for determining the preclinical screening of chemotherapeutic candidates [35]. In this study, we used two different doses to treat EAC-induced mice in the intraperitoneal cavity: 100 and 200 µg/kg body weight/day/mouse for 5 consecutive days. The maximum cell growth inhibition of 88.45% was found after treatment with 200 µg doses; while 100 µg doses of the test compound inhibited 83.78% cell growth (*p* < 0.01). The study observed treatment with [Ni(II)L](ClO_4_)_2_ at 200 µg and 100 µg/kg/day/mouse (i.p.) doses significantly increased 52.63% (MST 38.67 ± 1.76) (*p* < 0.01) and 36.84% (MST 34.67 ± 0.88) lifespan (*p* < 0.05) and reduced tumor weight (% RTW) 47.83% and 41.30%, respectively, compared to untreated EAC-bearing mice. Furthermore, the control group (EAC-bearing) showed a 63.88% increase in body weight by day 20 compared with normal mice, while mice treated with the test compound at 200 µg and 100 µg/kg doses (i.p.) showed only 31.58% (*p* < 0.01) and 38.57% (*p* < 0.05) increase, respectively, indicating a significant anticancer effect of [Ni(II)L](ClO_4_)_2_. Comparatively, metal-based complexes such as Cisplatin (Platinum-based) exhibit 49–85% tumor growth inhibition and 25–45% lifespan extension with nephrotoxic side effects [36–38], Ruthenium-based compounds exhibit 82%−88% tumor growth inhibition and 45% increase of lifespan in similar models [27], copper-based complexes show 60%−85% tumor growth inhibition and 51% lifespan extension (MST 28 days) [39, 40], Zinc complexes show 45% lifespan extension [39], suggesting a superior efficacy on tumor growth inhibition and lifespan extension of [Ni(II)L](ClO_4_)_2_.

**Fig. 7:**
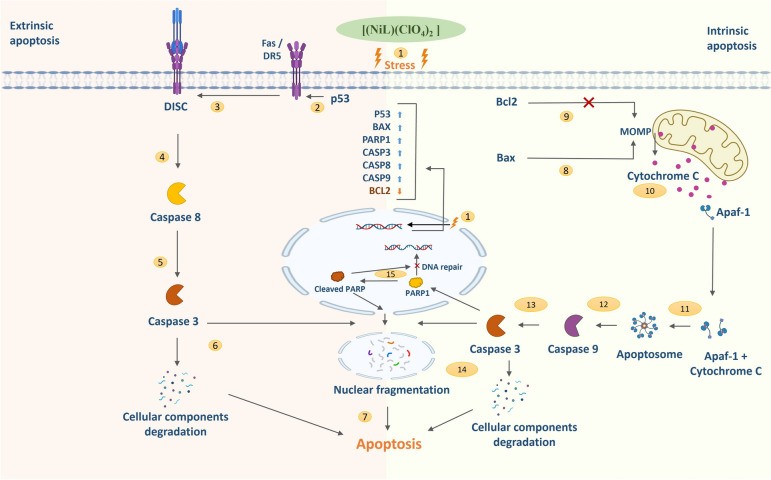
The possible mechanism of anticancer activity of the complex. In brief, **1** the compound induces intrinsic and extrinsic apoptotic pathways by upregulating pro-apoptotic genes (p53, BAX, PARP1, CASP3, CASP8, and CASP9) and downregulating anti-apoptotic gene BCL2 through internal stress signal. **2** In the extrinsic apoptotic pathway, p53 stimulates the increased expression of death receptors (Fas and DR5) on a cellular surface. **3** The death receptors bind with their ligand (FasL and TRAIL) to form Death Inducing Signaling Complex (DISC). **4** Formation of DISC induces the activation of caspase-8. **5** The inactive zymogen is activated as caspase-3 directly by caspase-8. **6** Activation of caspase-3 leads to the cleavage of various intracellular substrates and causes DNA fragmentation. **7** Cleavage of cytoskeletal protein and DNA through activated caspase-3, ultimately promoting apoptosis. **8** In the intrinsic apoptotic pathway, p53 induces the expression of Bax that causes mitochondrial outer membrane permeabilization (MOMP). **9** Also, p53 downregulates the Bcl2 that inhibits the MOMP formation. **10** MOMP results in the release of cytochrome C from the mitochondrial intermembrane to the cytosol. **11** Cytochrome C binds with apoptotic protease activating factor-1 (Apaf-1), resulting in the formation of the apoptosome complex. **12** The apoptosome converts procaspase-9 into activated caspase-9. **13** Caspase-9 cleaves inactive zymogen (procaspase-3) and activates caspase-3. **14)** The activated caspase-3 degrades the cellular components and causes nuclear fragmentation. **15)** Poly (ADP-ribose) polymerase-1 (PARP1) is an enzyme involved in the repair of damaged DNA that is cleaved by caspase-3 resulting in the inhibition of DNA repair. An excessive expression of PARP1 results in the cellular energy depletion and finally promotes apoptosis

To find out the molecular mechanism of [Ni(II)L](ClO_4_)_2_ in apoptosis induction, the study examined key apoptosis regulatory gene expressions such as *p53, BAX, PARP1, CASP3, CASP8, CASP9,* and *BCL2* through RT-PCR analysis. p53, a guardian of the genome, is a crucial tumor suppressor that responds to stress and DNA damage. p53 involves the engagement of death receptors such as Fas, DR5 (Death Receptor-5), and PERP to form DISC (Death-Inducing-Signaling-Complex) that lead to activation of CASP8 by a series of cascades which turn apoptosis through extrinsic pathway [41, 42]. A pro-apoptotic member of the Bcl2 family, BAX is transcribed by p53 and promotes mitochondrial outer membrane permeabilization (MOMP) that leads to the release of cytochrome c and triggers the formation of apoptosome complex that activates CASP9 and promotes the activation of CASP3 that carried out death program by cleaving various cellular substrates [43, 44]. The anti-apoptotic protein BCL2 inhibits BAX activity and prevents cytochrome c release by stabilizing the outer mitochondrial membrane. Low BCL2 expression makes it easier for BAX and pro-apoptotic proteins to damage the mitochondrial membrane, which promotes apoptosis [45]. As apoptosis progresses, active CASP3 cleaves PARP1 (Poly ADP-ribose polymerase-1), which serves as a marker of apoptotic cell death. Overexpression of PARP1 triggers energy depletion that drives the cell toward parthanatos, a type of cell death [46–48]. The study observed a significant pro-apoptotic response through the upregulation of pro-apoptotic genes such as p53, BAX, PARP1, CASP3, CASP8, and CASP9 with the downregulation of anti-apoptotic gene BCL2. Overall, these alterations in gene expression suggest the [Ni(II)L](ClO_4_)_2_ triggers both extrinsic (death receptor) and intrinsic (mitochondrial) apoptotic pathways, which makes it a potent inducer of apoptosis and a viable cancer therapeutic option. In comparison with other metal-based chemotherapeutic compounds such as platinum-based (cisplatin) trigger intrinsic mitochondrial apoptosis pathway via induction of BAX and BAK through p53 dependent pathway [49]; Ruthenium complexes upregulate p53, BAX, CASP3, CASP8, CASP9 and downregulate BCL2, induce both intrinsic and extrinsic pathway [27]; Copper complexes upregulate p53 induce caspase-3 activation apoptosis pathway [50, 51]; Gold complexes upregulate p53, BAX, CASP3, CASP9 and downregulate BCL2, induce intrinsic mitochondrial apoptosis pathway activation [52]; suggesting a unique property of both intrinsic and extrinsic apoptotic induction of [Ni(II)L](ClO_4_)_2_ proposed significant candidate for cancer therapy (Fig. 7).

In cancer chemotherapy, anemia and myelosuppression may occur caused by the decrease in hemoglobin and red blood cells [53, 54]. Most chemotherapeutic drugs have been characterized by myelosuppression, neutropenia, and thrombocytopenia by affecting blood cell production in different ways [55]. In this concern, hematological parameters of EAC-induced mice and treated EAC-induced mice with [Ni(II)L](ClO_4_)_2_ were examined. The study found, in comparison with normal mice, EAC-bearing mice exhibited lower hemoglobin levels (% of Hb) and fewer red blood cells (RBC), but higher total white blood cell counts (WBC). However, in EAC-induced mice with administration of test compound at doses of 100 µg and 200 µg/kg body weight/day/mouse (i.p.) for 10 constitutive days, hematological parameters moderately normalized, with a dose-dependent manner. This indicates [Ni(II)L](ClO_4_)_2_ has no host anemic and myelosuppressive effect.

In addition, this study investigated the host toxic effects of [Ni(II)L](ClO_4_)_2_ by examining hematological and biochemical parameters. Hematological profiles such as RBC, WBC, and hemoglobin serve as crucial indicators in evaluating the possible toxicity of the test compound to the body’s immune system, oxygen transport, blood-forming organs, and physiological stress [56, 57]. For example, RBC count can determine anemia due to bone marrow suppression (myelosuppression), hemolysis (destruction of RBC), and dehydration [58, 59]; WBC count may determine immune response, immune suppression, inflammation, or stress response (leukocytosis), bone marrow suppression (leukopenia) [60, 61]; hemoglobin concentration can determine oxygen carrying capacity, bone marrow suppression or hemolysis (anemia) [62, 63]. This examination found a moderate increase of WBCs during the treatment period, which normalized within two weeks after the termination of treatment, indicating a slight inflammation in response to the administration of [Ni(II)L](ClO_4_)_2_. This examination did not find any significant change in RBC count and hemoglobin concentration, indicating healthy bone marrow function, efficient oxygen transportation, and balanced blood generation.

Furthermore, the examination of biochemical parameters provides an extensive overview of metabolic health and organ function [64–67], such as SGPT and SGOT as indicators for liver health, whereas elevated level indicates stress and liver damage [64]; glucose levels as indicators for insulin response and pancreatic function [67]; cholesterol levels as indicators for lipid metabolism and cardiovascular health [66]; and creatinine levels as indicators for kidney function [65]. This examination found a slight increase of SGPT and creatinine levels during the administration period of the [Ni(II)L](ClO_4_)_2_, which normalized gradually after the termination of treatment indicating a short-term with no long-term toxic effect on liver and kidney function. This examination did not observe any significant change in SGOT, glucose, and cholesterol indicating healthy pancreatic and cardiovascular health with efficient insulin response and lipid metabolism. In comparison with other metal-based chemotherapeutic drugs such as Cisplatin causes nephrotoxicity, ototoxicity, immunosuppression, and severe myelosuppression [68]; Ruthenium complexes cause mild hepatotoxicity, nephrotoxicity, and genotoxicity at higher doses [69]; Copper complexes cause hypoxia, angiogenesis, regulation of glycolysis and induce metastasis at higher doses [70]; Gold compounds exhibit aplastic anemia, dermatitis, glomerulonephritis, renal damage, stomatitis, and bone marrow suppression [71]; Nickel complexes cause hypoxia, lung inflammation, and oxidative DNA damage [72]. Arsenic trioxide as a therapeutic agent causes mitochondrial dysfunction that may lead to diabetes, stroke, hypertension, and heart disease [73, 74], emphasizing the test compound [(NiL)(ClO_4_)_2_] complex’s therapeutic potential with low host toxicity of minimal short-term liver and kidney stress. In addition, the tested compound did not exhibit major damage at the tissue levels of major organs of the animal in histopathological analysis. It causes minor deformation in animal organs, which could be recovered after some time of treatment. These results indicate that the compound has no chronic effects on cellular structure and organ functions.

Limitations of the current study: This study has some limitations. For example, we only measured gene expression at the mRNA level and did not confirm the protein changes by methods like Western blot or ELISA. Also, we used only one cancer cell line (MCF7) for in vitro testing and did not use any normal human cell line to check the safety of the compound. Although we tested normal mice for toxicity, more studies are needed to confirm its safety in normal human cells. In addition, we did not study the long-term effects or how the compound behaves in the body over time. These points can be explored in future research.

## Conclusion

Overall, the [Ni(II)L](ClO_4_)_2_ demonstrated promising anticancer properties, including significant cytotoxicity, tumor cell growth reduction by dual activation of intrinsic and extrinsic apoptotic pathways, and life span extension that is referred to as a flexible option for overcoming single-pathway drug resistance. In addition, lower host toxicity implied a safer substitute for traditional chemotherapeutics after further investigation of several studies such as determining anticancer efficacy on other cancer types such as lung, colon, prostate, and other cancers, followed by evaluating the effects on cell migration, invasion, and angiogenesis and studying the enhancing efficacy of [Ni(II)L](ClO_4_)_2_ combination with existing chemotherapeutics or targeted therapies.

## Data Availability

No datasets were generated or analyzed during the current study.
